# BETting on combination to overcome PARPi resistance

**DOI:** 10.18632/oncotarget.21446

**Published:** 2017-10-01

**Authors:** Youyou Zhang, Chi V. Dang, Lin Zhang

**Affiliations:** Youyou Zhang: Center for Research on Reproduction and Women’s Health, University of Pennsylvania, Philadelphia, PA, USA; Department of Obstetrics and Gynecology, University of Pennsylvania, Philadelphia, PA, USA

**Keywords:** BET inhibitor, PARP inhibitor, targeted therapy, MYC, tumor immunity

PARP Poly (ADP-ribose) polymerase (PARP) is an enzyme family that post-translationally modifies its target proteins by conjugating polymeric chains of ADP-ribose (poly-ADP-ribosylation) during a number of cellular processes including DNA repair, transcription, translation, cell signaling, and cell death. The foundational works in 2005 demonstrating synthetic lethal interaction between loss of BRCA function and PARP inhibition have led to the approval of PARP inhibitors (PARPis) for clinical use. Most recently, the FDA has approved PARPis as monotherapy for women with advanced ovarian cancer. It has also shown promising clinical activity in breast and prostate cancers, especially in tumors with defective DNA repair via homologous recombination (HR). However, HR-deficient tumors represent only a small fraction of cancers, which restricts the clinical application of PARPi as monotherapy. Moreover, tumors initially HR-deficient commonly acquire HR-proficiency after PARPi treatment. Therefore, intrinsic and acquired resistance is a major clinical problem for PARPi in cancer care. Development of drug combination strategies to selectively impair HR in cancer cells and subsequently sensitize HR-proficient cancers to PARPi would lead to novel clinical applications for cancer treatment.

Given that agents that repress transcription of genes involved in HR have been reported to enhance response to PARPi, it has been hypothesized that modulation of epigenetic regulators may impair expression of genes in HR, subsequently render HR-proficient tumors HR-deficient, and thus sensitize them to PARP inhibition. Recently, Yang *et al.* performed a drug combination screen using a FDA approved PARPi, olaparib, and 20 well-characterized epigenetic drugs targeting seven classes of epigenetic regulators in a triple-negative breast cancer cell line [1]. Interestingly, all three bromodomain and extraterminal domain inhibitors (BETis: JQ1, OTX015 and I-BET762) showed strong synergistic effects with olaparib. Given that well-ordered, deep, hydrophobic pockets in BRDs provide a highly favorable locus for small molecule compounds, a number of BETis have been developed and evaluated in multiple preclinical cancer models. In this study, functional assays further demonstrated that repressed BET activity reduces HR and subsequently enhances PARPi-induced DNA damage in cancer cells. Chemical inhibition or genetic depletion of BET proteins impairs transcription of *BRCA1* and *RAD51*, two essential genes in HR. These results provide strong rationale for clinical application of PARPi in the setting of combination with BETi to treat both cancers with *de novo* resistance to PARPi therapy and cancers with acquired resistance.

Multiple mechanisms of synthetic lethality between PARP inhibition and HR deficiency have been reported, including suppression of base excision repair (BER) and activation of non-homologous end joining (NHEJ). PARPi leads to BER dysfunction that induces persistent single-strand breaks, which are converted to double-strand breaks (DSBs) by collision with replication machinery. Consequently, the inability of HR-deficient cells to adequately repair DSBs results in cell death. Meanwhile, due to PARP also preventing NHEJ components from binding to DNA damage sites, in the absence of HR and PARP activity, activated NHEJ (an error-prone repair) aberrantly processes DNA and induces chromosomal instability, leading to cell death. It has been demonstrated previously that MYC increases transcription of genes in the HR pathway including *BRCA1* and *RAD51* [2], and overexpressing MYC inhibits NHEJ activity by repression of Ku80/70 DNA binding and DNA-PKcs activity [3]. Thus, BETi may suppress HR and enhance NHEJ, thereby sensitizing initially HR-proficient cancer cells to PARP inhibition through two related mechanisms: BETi directly represses transcriptional elongation of *BRCA1* and *RAD51*, thus preferentially inhibiting expression of genes critical to HR; additionally, in MYC-hyperactivated conditions, suppression of MYC by BETi further represses BRCA1/RAD51 expression and increases NHEJ activity, synergistically sensitizing cells to PARP inhibition. Given a large percentage of cancers (>30%) with accumulated MYC expression, further characterization of the roles of MYC in PARPi and BETi combination will significantly impact the clinical development of BETi/PARPi combination therapy by defining whether MYC-hyperactivated patients are more sensitive to BETi/PARPi combination treatment; and explaining why cancer cells are more sensitive to BETi/PARPi combination compared to normal cells in which MYC is expressed at a physiological level.

Targeted therapies may also affect aspects of tumor immunity, e.g., T cell function, T cell trafficking /infiltration, or tumor antigenicity. It has been reported that BETi enhances anti-tumor T cell persistence/function [4], represses PD-L1 expression [5], attenuates Treg function [6], and influences inflammation; however BETi also blocks differentiation of T helper 17 cells (T_H_17) [7] that promote protective anti-tumor immune responses. Meanwhile, although PARPi may promote local antigen release, it also upregulates PD-L1 expression in tumor cells [8]. Therefore, how the anti-tumor immune system responds to BETi/PARPi combination therapy is a complicated and important question for further clinical development of this combination.
